# Challenges and Opportunities in Scaling up Architectural Applications of Mycelium-Based Materials with Digital Fabrication

**DOI:** 10.3390/biomimetics7020044

**Published:** 2022-04-14

**Authors:** Selina Bitting, Tiziano Derme, Juney Lee, Tom Van Mele, Benjamin Dillenburger, Philippe Block

**Affiliations:** 1 Block Research Group, Institute of Technology in Architecture, ETH Zurich, Stefano-Franscini-Platz 1, HIB E 45, 8093 Zurich, Switzerland; bitting@arch.ethz.ch (S.B.); van.mele@arch.ethz.ch (T.V.M.); block@arch.ethz.ch (P.B.); 2 Digital Building Technologies, Institute of Technology in Architecture, ETH Zurich, Stefano-Franscini-Platz 1, HIB E 23, 8093 Zurich, Switzerland; derme@arch.ethz.ch (T.D.); dillenburger@arch.ethz.ch (B.D.)

**Keywords:** mycelium, architecture, structural design, computational design, digital fabrication, additive manufacturing, subtractive manufacturing, circular economy

## Abstract

In an increasing effort to address the environmental challenges caused by the currently linear economic paradigm of “produce, use, and discard”, the construction industry has been shifting towards a more circular model. A circular economy requires closing of the loops, where the end-of-life of a building is considered more carefully, and waste is used as a resource. In comparison to traditional building materials such as timber, steel and concrete, mycelium-based materials are renewable alternatives that use organic agricultural and industrial waste as a key ingredient for production, and do not rely on mass extraction or exploitation of valuable finite or non-finite resources. Mycelium-based materials have shown their potential as a more circular and economically competitive alternative to conventional synthetic materials in numerous industries ranging from packaging, electronic prototyping, furniture, fashion to architecture. However, application of mycelium-based materials in the construction industry has been limited to small-scale prototypes and architectural installations due to low mechanical properties, lack of standardisation in production methods and material characterisation. This paper aims to review the current state of the art in research and applications of mycelium-based materials across disciplines, with a particular focus on digital methods of fabrication, production, and design. The information gathered from this review will be synthesised to identify key challenges in scaling up applications of mycelium-based materials as load-bearing structural elements in architecture and suggest opportunities and directions for future research.

## 1. Introduction

As a consequence of its current linear economic model of “produce, use, and discard”, the construction industry is a significant contributor to global greenhouse gas emissions, destruction of natural habitat and production of industrial waste [1,2]. The industry’s reliance on a select few non-renewable materials, such as concrete and steel, puts environmental pressure on finite natural resources, which could eventually lead to their permanent depletion [3]. While the industry is increasingly shifting towards more renewable building materials, the long-term environmental impact of this accelerating growth in demand and rate of regeneration remains to be seen. This shift is spearheaded by mass engineered timber (MET) and engineered bamboo composite (EBC) that depend on additional natural resources such as soil and water, which consequently experience increases in demand [4]. The transition from extracting non-renewable resources to harvesting renewable ones alone does not necessarily guarantee that the resultant material has a fully circular life cycle. For example, synthetic adhesives are fundamental for the production of MET and alternative bio-based solutions still largely rely on the usage of chemicals. This hinders the commercialisation of fully sustainable, circular wood products, which in turn impedes a transition to more circular and environmentally conscientious material sourcing [5].

Similar trends can be observed with the emergence of bio-resins and bio-plastics. These serve as alternatives to their more traditional, petroleum-based counterparts. However, the production of these alternatives typically relies on a single commodity feedstock, such as corn or sugarcane. This causes a rise in demand for these feedstocks for industrial use, creating competition with existing stock for food supply and instigating complex socio-economic policy problems [6]. There are similar consequences for the rapidly growing demand for MET, which have the potential to intensify the current volume and rate of deforestation across the world. While this rising demand can be potentially addressed with a fast-growing and high-yield material such as bamboo in certain contexts, hardwoods such as oak that are used for the production of most commercially available MET have the highest potential for contributing to further deforestation [7]. Therefore, there is a considerable need to find new alternative materials that are not just naturally cultivated and harvested, but also produced with processes that repurpose waste streams and improve the reusability and recyclability at the end of their life cycle.

### 1.1. Mycelium as Pilot Material for Circular Construction

A more circular approach to sustainable materials considers using industrial waste such as silica fly ash or lignocellulosic agricultural waste such as rice hulls, saw dust, corn cobs or soybean stalks as ingredients for the production of new bio-based materials [8]. Most of these approaches focus on repurposing low-value waste material into admixtures for concrete. However, there are also processes that transform industrial by-products and agricultural waste into completely new, high-value materials. 

**Figure 1:**
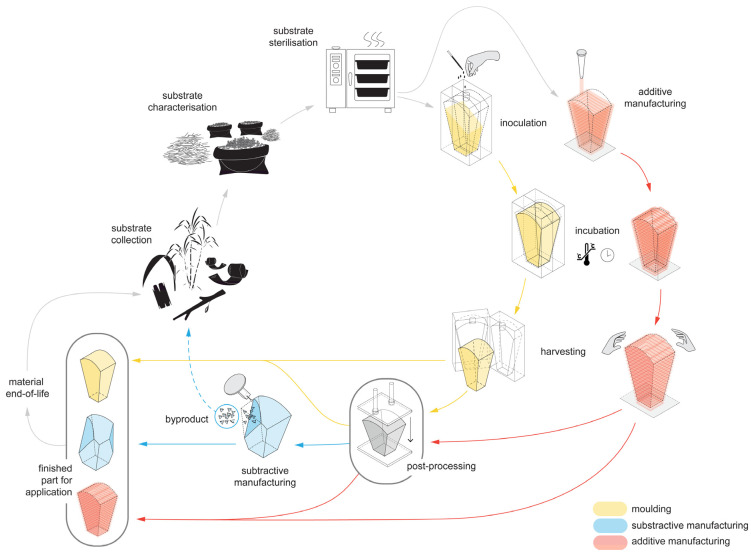
Circularity and workflow of current manufacturing processes applied to mycelium-based materials, adapted from [15,16,17].

Recent developments of renewable composites based on mycelium have demonstrated a tremendous amount of potential in repurposing industrial waste streams into a viable resource for producing more sustainable and circular materials (Figure 1). Mycelium is the vegetative part of fungi that consists of a dense network of micro-filaments called hyphae [9,10] that have the capacity to bind food, agricultural and industrial waste that have very little or no commercial value and convert them into higher-value composite materials with a wide range of potential applications. The success of startups specialising in mycelium-based research, production and applications has also shown how this material can be introduced into the current market to have an immediate economic impact and reward [11,12,13].

In addition to using low amounts of energy during production and having a high profile of biodegradability, mycelium-based materials are highly customisable throughout the growing and manufacturing processes. This enables production of mycelium-based materials with various properties, which can satisfy varying criteria from different disciplines, and be suitable for different applications [14]. In the construction industry, mycelium-based materials have already gained traction in non-structural applications, such as for thermal and acoustical panels, thanks to their naturally high insulative properties as well as their resistance to fire [15].

### 1.2. Problem Statement

Despite their advantages, applications of mycelium-based materials as load-bearing structural elements have been limited, primarily due to their low mechanical properties. Considering that the structural mass is the predominant contributor to the total embodied carbon of a building, structural applications of low-carbon materials such as mycelium have the potential to significantly improve a building’s environmental performance [18]. However, their comparatively low structural load capacity means that applications of mycelium-based materials need to be closely coupled with structurally informed geometry and appropriate digital fabrication methods.

The underutilisation of mycelium-based materials for large-scale architectural applications is also caused in large part by the monopoly of mycelium-related patents in the industry. These patents belong to a select few of the previously mentioned startups and prevent the distribution of knowledge to successfully mass-produce high-quality, standardised mycelium-based materials. The lack of generalised knowledge also leads to a lack of awareness by the public about the existence of these materials and a lack of confidence in large-scale applications beyond packaging alternatives and consumer products [19]. 

Furthermore, there is a disconnect between the industry and academia. Publications on new research and applications of mycelium-based materials tend to withhold information and data regarding the fungal species used, incubation parameters, substrate compositions, or detailed fabrication procedures, as a vast majority of the authors are affiliated with commercial companies [20].

There has been a recent surge of publications reviewing research and applications of mycelium-based materials in architecture, and while many have similarities, there is also a broad range of stances and scopes [4,15,19,20,21,22,23,24,25,26]. Most of these reviews focus on applications of mycelium-based materials for bespoke architectural prototypes and installations. However, this is a narrow scope that can lead to the exclusion of emerging innovations from other disciplines, such as mycology, microbiology, and material science as well as the fashion and textile industry. Additionally, the reviews tend to focus mostly on mycelium-bound composite materials, while lesser-known or less-documented implementations using pure mycelium have the potential to open new avenues of research and design applications in architecture.

### 1.3. Objectives

The main aim of this paper is to review the latest developments in research and applications of mycelium-based materials across multiple disciplines, including those that are not typically associated with the construction industry, with a focus on digital fabrication techniques. This review will investigate different typologies of mycelium-based materials at varying scales of interest, starting from small-scale commercial products and working up to larger architectural applications. The intent is to understand the scales at which mycelium-based materials tend to be implemented in the current state of the art. The key challenges that are hindering the scaling up of their applications then can be identified, along with the opportunities to overcome those challenges. Speculative strategies for scaling up applications of mycelium are then proposed in later sections, focusing on appropriate selection of material typology in conjunction with relevant fabrication and construction methods. 

### 1.4. Contributions and Outline

The paper is organised as follows.

In Section 2, we present a review of the latest advancements and applications of mycelium-based materials across a wide range of scales and disciplines. Methods of growing and processing different mycelium material typologies are discussed, followed by an overview of typical production and digital fabrication methods that are often used in literature. Finally, various applications of mycelium-based materials are examined, both at the scale of smaller commercial products and larger architectural prototypes and installations.

In Section 3, we synthesise the information gathered from the state of the art to identify the key challenges in scaling up applications of mycelium-based materials in architecture as load-bearing structural elements. For each challenge, we suggest opportunities and outline directions for future research with a focus on relevant computational structural design and digital fabrication techniques.

## 2. State of the Art

This section on the state of the art is organised in three subsections: mycelium material typologies, fabrication methods, and applications.

### 2.1. Mycelium Material Typologies

There are two primary methods for developing engineered mycelium-based materials: pure mycelium materials (PMM) and mycelium-bound composites (MBC). The first refers to creating a mycological biopolymer by harvesting a liquid culture of pure mycelium. The second refers to a bio-composite wherein the hyphal network of the mycelium binds lignocellulosic substrates. In both approaches, the air contained within the mycelial network or between the loosely packed substrates and mycelia matrix results in a low-density material with foam-like properties [15].

**Figure 2:**
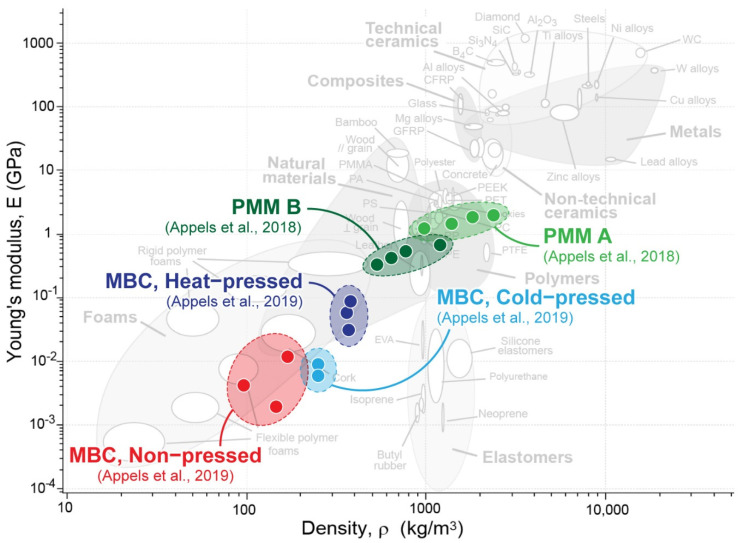
A comparison of mycelium-based materials to standard materials, with data adapted from [30,31]. Underlying Ashby plot created using CES EduPack 2019, ANSYS Granta © 2020 Granta Design.

PMM and MBC have material characteristics similar to polystyrene and polyurethane foams, respectively [19] (see Figure 2). These materials, without post-processing, are primarily suitable for non-structural applications and, particularly in the case of MBC, can be used as excellent thermal and acoustic insulators. The mechanical properties of these materials, described by several authors [15,27,28,29], vary depending on the fungal strain chosen for the inoculation, the origin and type of the substrate, and the growth conditions [30].

Substrates play a fundamental role in the final material properties. Their initial weight, porosity, and nutritive profile determine the consequent thermodynamic, physical, and mechanical behaviour of the biomaterial. As reported by Islam et al. [10] and Girometta [15], the balance between fungal and plant biomass can drastically determine the density of the composite, especially in the case of MBC. Implementations which prioritise material strength typically value higher density. However, in addition to density, rendering the material inert and post-processing the material have the possibility to increase or decrease the mechanical strength of both PMM bio-polymers and MBC bio-composites.

#### 2.1.1. Pure Mycelium Materials

In accordance with the definition described by Vanderlook et al. [16], PMM are mycelial bio-polymers that do not contain any lignocellulosic substrates. They are characterised solely by the biological properties of the fungal species, source of nutrients, and growing conditions. The notable feature of these materials is their reliance on the use of liquid mycelium cultures [32]. The basic method for producing these mycelial bio-polymers starts with several containers, each of which defines the cavity that contains a soft scrim, the nutritive substrate, and the desired fungal strain. These containers are subsequently placed into a closed incubation chamber with directed airflow at target humidity level and temperature [33,34,35].

The use of this specific culturing method allows for the rapid and consistent production of mycelial biomass and a precise control of the growth process. Thanks to its flexible foam-like properties, PMM can be produced and post-processed to be suitable for the textile, footwear and paper making industry or as green alternatives to polymeric foams such as expanded polystyrene and materials where flexibility is preferred over rigidity [36] (Figure 2). Within the various steps of growing and processing these mycelium-based materials, technology and fermentation can have a crucial influence on the resultant material as well as in scaling up its production. For example, the use of bioreactors fulfils the need for a controlled environment with the potential of producing large quantities of material [37]. Research from the VTT Technical research centre in Finland showed the potential of mass producing the mycelial bio-polymer through the creation of a continuous PMM production using bioreactor fermentation [38].

#### 2.1.2. Mycelium-Bound Composites

MBC are bio-composites characterised by the growth of a fungal strain on substrates comprising discrete particles of organic or inorganic origin [30]. During the growth process, mycelium digests the nutrients contained in the substrate, penetrating the fibres and developing a tight network of hyphae. These hyphae bind the substrates together, ultimately transforming the mixture into a self-supporting, consolidated mycelial mass [15,39]. MBC can be produced and post-processed to create tough, pliable material with mechanical properties similar to those of wood and cork [30] (Figure 2).

The balance between the mass of the substrate and the mycelial mass enables the creation of versatile composites with tuneable density and porosity. Most of the research investigating the physical properties of MBC explore the influence of various types and sizes of substrate fibres, as well as the type of inoculation (grain spawn or liquid culture) [40,41]. The substrates used to maximise the growth of mycelium consist of lignocellulosic materials coming from agricultural crop waste such as, but not limited to, cotton, corn, flax, hemp, and wheat. These substrates, depending on their nutritional profile, affect the density of the material, as a higher proportion of grains typically corresponds to a higher material density [24,42]. Depending on the desired material profile and field of application, the use and customisation of substrates can be tailored to achieve the desired properties in the final material.

As suggested in the patent of Schaak Damen [43] from the company Ecovative, the production of MBC can also implement a more complex process that utilises a bacterial species in combination with a fungal species, non-nutritional substrates, and additional nutritive materials. In this approach, the bacteria, through its metabolic process, provides mechanical properties to the bio-composite material, and the fungal species binds the bio-composite.

### 2.2. Fabrication Methods

The simplest method for fabricating mycelium-based materials is to grow it in a mould with a predefined geometry. There are two primary fabrication methods that can be applied afterwards: subtractive and additive manufacturing. 

Subtractive manufacturing uses pre-grown MBC components or panels, which are then altered via cutting, milling or some other subtractive process. This transforms MBC into a multitude of products and architectural building components. Subtractive processes can be applied to mycelium materials that are either alive or rendered inert, and are typically associated with discretised forms, rigid blocks, and panelised mycelium-based products. 

Alternatively, additive manufacturing describes the methods that are applied prior to either MBC or PMM during the growth phase. This fabrication methodology needs to be carefully integrated into the production pipeline as it can directly influence the growing and harvesting processes of the material (i.e., preparation, inoculation, and incubation). This results in different products with different material characteristics. Typically, additive manufacturing is associated with the production of low-density mycelium products, or large in situ architectural components.

#### 2.2.1. Moulds

The methodology typically used to produce MBC includes three main steps that come after the preparation and the sterilisation of the selected substrate: (1) inoculation of the substrates in a controlled environment; (2) incubation of the material for a period ranging from 7 to 30 days, depending on the fungal species under specific humidity, temperature, and air conditions; and (3) transfer of the colonised mycelium material outside the controlled environment and exposure to high heat to halt and terminate the growing process. Due to the high sensitivity to humidity, temperature, and air quality, the first two steps often rely on containing the mixture in pre-defined moulds. These moulds can be produced with customised geometry, then sealed to maintain a constant controlled environment for the fungal propagation [40]. Reproducibility of laboratory facilities and mass customisation are crucial steps for large-scale production and application of MBC produced with moulds [44].

#### 2.2.2. Subtractive Manufacturing

**Figure 3:**
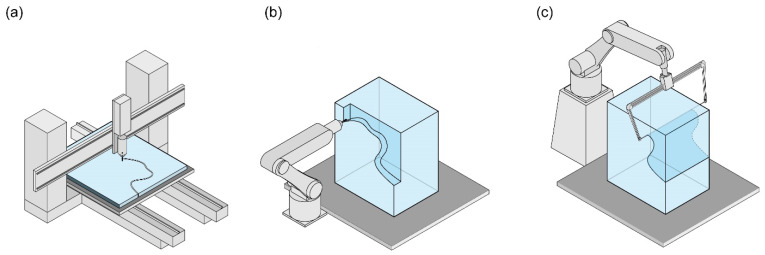
Typical subtractive manufacturing techniques: (**a**) milling (cutting) flat, panel-like specimen; (**b**) milling (carving) volumetric specimen; and (**c**) wire-cutting volumetric specimen.

Subtractive manufacturing (SM) refers to processes that involve removal of material. In a subtractive manufacturing workflow using mycelium-based materials, modules of prefabricated MBC are milled, wire-cut or undergo some other subtractive process. These modules can be products such as insulation, packaging or acoustical insulation, or they can be assembled into larger structures or pavilions. Subtractive methods in combination with MBC are not widely implemented due to several disadvantages, such as the need to sterilise equipment and the inconsistent fibrous nature of the material being difficult to work with. However, methods such as milling [45,46] and wire-cutting [17] have recently shown promise in spite of them. The main advantage of utilising this fabrication method is the elimination of the necessity for complex moulds, as geometric complexity can be added by machining away material instead (Figure 3). 

**Milling**

Milling is a method that can be applied not only to panel-like geometries but also to more volumetric ones (Figure 3a,b). Milling materials such as medium-density fibreboard (MDF), oriented strand board (OSB), or polystyrene produces dust and debris as a by-product, which is typically discarded as waste. The circular nature of mycelium-based materials would allow these by-products to be re-used. MBC can be milled before or after autoclaving or applying a post-processing method such as pressing. However, there are some risks and complications in doing so. In the case where mycelium is milled while the mycelium hyphae are still growing, additional considerations must be made to prevent contamination, such as sterilising all equipment and milling in a closed, controlled environment. If rendered inert, contamination is no longer a risk.

**Figure 4:**
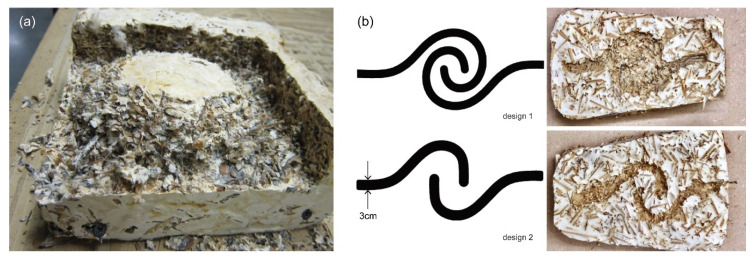
Two examples of MBC grown in a mould, rendered inert, and CNC milled thereafter Reproduced and reprinted with permission from: (**a**) Peek; 2021 [45]; and (**b**) Lazaro et al., Proceedings of the UbiComp/ISWC; 2019 [46].

Examples of milling MBC after autoclaving are exhibited in Figure 4 and show how the substrates in the MBC have ripped up during the milling process. This results in an undesirable surface finish and inadequate geometric precision. Furthermore, because MBC are anisotropic materials [47] with a structural performance that is stochastic [48], this method of fabrication then has the potential to aggravate the structural performance and material characteristics of the block in an unpredictable manner. Some fungal strains, for example, develop a skin (seen in white in Figure 4), and this skin increases the material’s compressive strength as well as its water repellence [32]. The removal or harming of this skin is therefore disadvantageous to the mechanical properties of MBC.

**Wire-cutting**

While wire-cutting (Figure 3c) also produces excess material, it is quicker than milling, which is an advantage. Elsacker et al. [17] highlights the potential of this subtractive method in combination with MBC, where both living and inert MBC blocks are cut with an abrasive wire cutter. When a living block is cut, both halves are incubated further after cutting to allow the cut surface to grow into a thickened outer coating or “skin”, which is advantageous from an architectural perspective in terms of aesthetics as well as the material properties. This shows the advantage of designing geometries with wire-cutting, as the excess material can be planned to become another inverse module to be used in construction. These blocks are later used as an insulative formwork for concrete, which is elaborated upon in Section 2.3.1. The advantage of this approach is in not creating excessive amounts of dust and the potential to incorporate both the “positive” and the “negative” of the cut specimen.

#### 2.2.3. Additive Manufacturing

In the past decade, additive manufacturing (AM) has been widely used to produce three-dimensional products following a layer-by-layer, gradual material deposition method. The growing demand for the use of this technology refers directly to its advantages of being a form of rapid prototyping with the capacity of producing geometrically complex parts without added cost [49]. This led to the expansion of the technology towards the use of numerous materials across different industries from the automobile, aerospace, construction, medical, and food industries, to many others. AM can be divided into various categories that depend on the type of process and material state. These include liquid-based, solid-based, powder-based, and gas-based processes [50] as well as new subcategories that have emerged from the combinations of two or more different types of AM such as slurry-based 3D printing.

**Figure 5:**
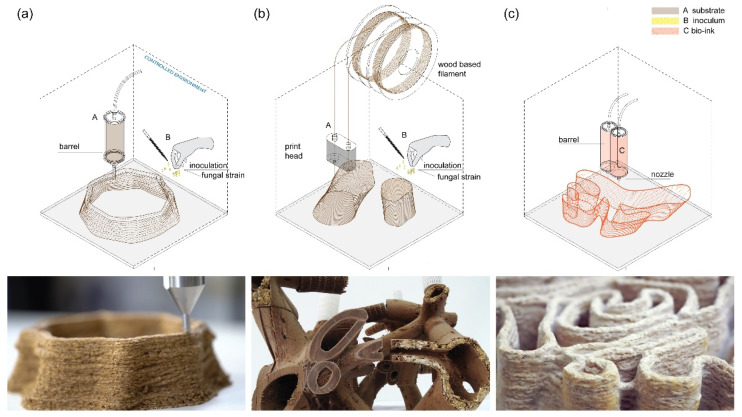
Current additive manufacturing approaches for mycelium-bound composites: (**a**) substrate core deposition [52] (Julia Krayer and Fraunhofer UMSICHT © 2022); (**b**) filament-based scaffolds [55] (reprinted with permission from Alima et al., Proceedings of the 2021 DigitalFUTURES; 2022); and (**c**) bio-inks [59] (reprinted with permission from Goidea et al., Pulp Faction: 3d Printed Material Assemblies through Microbial Biotransformation; published by UCL Press, 2020).

AM applied to mycelium-based materials refers to the creation of mycelium-based composites that integrate lignocellulosic substrates with the inoculation of a fungal strain during or after the manufacturing process. Due to their new and unique processes, various applications of AM to mycelium-based materials represent extensions of current advancements into bio-printing. This technique is mainly used in tissue engineering and regenerative medicine, and consists of manufacturing with living microorganisms, scaffolds, and the transformation of materials into complex living tissue [51]. While the differentiation between current approaches of AM is somewhat flexible, AM approaches that are used with mycelium-based materials can be largely categorised into three main areas of investigation: substrate core deposition; filament-based scaffolds; and bio-inks (Figure 5).

**Substrate core deposition**

This fabrication methodology can be associated with Direct Ink Writing (DIW), a 3D printing technique in which a paste-like material is deposited in a layer-by-layer fashion. Most of the experimentations found in literature use this methodology to develop an extrudable paste made of lignocellulosic material for the fabrication of a scaffolding structure. The deposition process is usually followed by the inoculation of the substrate core scaffold with a selected fungal strain. These approaches often result in small-scale vase-like structures, as seen in the work of Fraunhofer UMSICHT [52]. In the case of the experimentation developed at the IAAC in Barcelona [53], the use of clay as the main substrate characterised the additive manufacturing process. The resulting lattice structure was printed and designed to be ideal for the post-inoculation process. 

**Filament-based scaffolds**

This fabrication approach is an extension of widely available fused deposition modelling (FDM) printers, capitalising on the research of specific filaments that contain sufficient nutrients to enhance mycelium interfacial bonding. The filament is printed first and thereafter inoculated with the desired fungal strain. These opportunities came along with the commercialisation of the first wooden filaments on the market, such as Laywoo-D3 or Growlay [54]. Due to their composition and capacity to absorb water, these filaments may serve as a means for the growing plants, or in this case, fungi. Investigations of this application are mostly academic and emphasise the role of 3D printed scaffolds. Notable experiments in this area come from the PhD research of Nicole Alima [55] on bio-scaffolds, and from the Myco Mensa table developed by Richard Beckett [56]. These experimentations are characterised using generative design tools and less on the definition of material properties and performance.

**Bio-inks**

This material typology integrates an organic substrate, a carrier, and living cells directly into an extrudable paste or mixture with shape retention properties. The carrier is normally a biopolymeric gel that acts as a molecular scaffold [57]. Promising investigations in this area come from Eugene Soh et al. [58] and the group of Professor H. La Ferrand from Nanyang Technological University of Singapore. The publication suggests an experimental framework that integrates (a) the direct experiment with new substrates such as bamboo fibres, (b) with the selection of a specific fungal strain (i.e., *Ganoderma lucidum*), and (c) the use of additives, such as chitosan-based solutions, are used to increase the workability and buildability of the extrusion paste. In this research, the carrier acts as a provisional binder for the substrate and combines the fabrication and inoculation into one single step. Goidea et al. [59] use a combination of clay and organic substrates for the creation of an extrudable inoculated paste. These experimentations are interested in the production of large-scale, load-bearing demonstrators. Similar is the case in 2016 of the project Bio Ex-Machina conducted by Officina Corpuscoli, Utrecht University, and the European Space Agency’s Advanced Concepts Team (ESA/ACT) [60].

### 2.3. Applications

**Figure 6:**
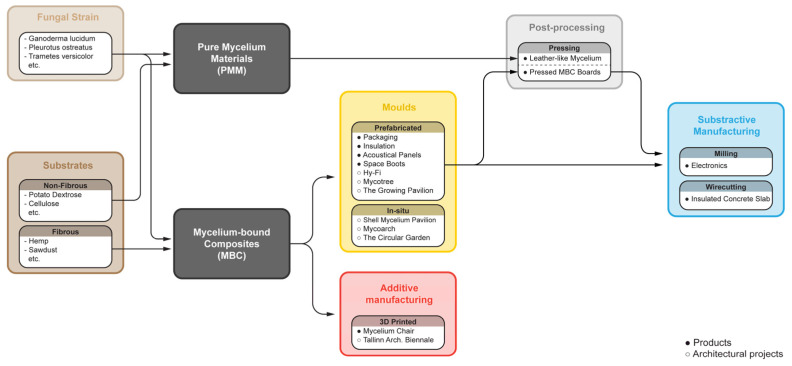
Overview of mycelium-based material typologies and applications.

The applications of mycelium-based materials to be reviewed are sorted into two categories based on the scale of the application: products (small) and architectural projects (large). Some examples of implementations that are in the products category are items aimed at consumers, such as furniture, jewellery, clothing, or building components such as interior finishing products and insulation. The architectural projects section showcases assemblies composed of mycelium-based materials that are larger in scale than a singular module or piece of furniture. The products and architectural applications are summarised and categorised in Figure 6, according to the typology of mycelium-based material used and the fabrication method applied.

#### 2.3.1. Products

The following products are small-scale applications of the mycelium material typologies presented in Section 2.1.

**Fashion**

There are multiple types of mycelium-based textiles that are relevant to the fashion industry, applicable to items such as clothing and shoes. For example, flexible mycelium foams can be used as an inner insulation layer to fabric as an alternative to down feathers [61]. The foam is grown by filling macroscopic void spaces with elements such as agar beads, which are incorporated into the mycelial matrix during the growth process and then removed later through exposure to high heat. This does not damage the mycelial network itself but does destroy the filler elements and results in a flexible foam-like material. This product is patented by Ecovative [62], and demonstrates how influential post-processing of PMM is on the resulting material. The production of a leather-like mycelium material is an elaboration of this process.

The development of mycelium-based substitutes for leather utilises liquid culture to produce sheets that are then manipulated to be tanned and turned into bags, clothes, etc. The rolls are produced as flexible mycelium foams, then pressed and further manipulated to have leather-like material properties. One company that is utilising this technology is Bolt Threads, which engineered Mylo in tandem with Ecovative, who are working to bring these products to the consumer market. The process used to produce Mylo results in an non-biodegradable product that is not plastic-free and is certified 50–85% bio-based [12]. Companies such as Adidas, Lululemon, and Stella McCartney have designed numerous products with Mylo, although in most cases they are not yet sold in stores.

Alternatively, a recent article by E.R. Kanishka B. Wijayarathna explores a fully biodegradable mycelium-based leather alternative, which is made of fungal biomass cultivated on bread waste. Glycerol and a bio-based binder were used in the treatment of this leather-like material, which was successfully used to create a prototype phone pouch as well as a coin wallet. Sheets of the untreated material with only glycerol post-treatment resulted in a tensile strength of 7.7 MPa, whereas sheets of the untreated material and tannin-treated material with both glycerol and binder treatments led to tensile strengths of 7.1 MPa and 6.9 MPa, respectively [63]. These two variations of mycelium-based leather substitutes demonstrate the influence that post-processing methods have over material characteristics. 

A more futuristic application of mycelium is seen in the proposed design for mycelium space boots. The intent is to reimagine a moon boot into something that could be manufactured entirely aboard a space shuttle. The final design achieves this, with basically only human sweat and a few fungus spores, ideal for a seven-month trip to Mars with limited space. This is an implementation that displays how design goals have a great influence on mycelium products, as well as how it is possible to push the boundaries of what is possible with these materials [64]. 

**Furniture**

One of the most notable and talked-about products utilising mycelium is the chair designed in 2003 by Eric Klarenbeek [65]. This project is one of the first implementations of 3D printing technologies with MBC. The process of fabrication is characterised by the creation of a hollow 3D printed scaffold acting as temporary mould for the substrate deposition. On the contrary, Myco Mensa, a table designed by Richard Beckett [56], integrates robotic fabrication and generative design approaches with the direct deposition of organic matter. Another product is Mycosella, a project presented in 2019 from a graduate from Newcastle University, which investigates the production of a series of stools and chairs that integrate MBC with other materials (i.e., wood, steel) in different manners [66]. 

**Packaging**

One of the first mycelium-based products to reach the consumer market is Ecovative’s mycelium packaging. This product utilises a growth mixture of hemp hurd and a fungal strain [61]. After use, mycelium packaging can be broken up and composted without any additional steps to naturally decompose. While this foam alternative is a significant improvement from conventional synthetic packaging material in terms of environmental impact, the main disadvantage is the increased weight and bulk. Where conventional polystyrene foam has an approximate density of 10–50 kg/m^3^, an alternative mycelium packaging can have a density between 55–210 kg/m^3^. Since shipping costs are typically determined by weight, mycelium packaging is not yet able to fully replace the traditional polystyrene foams [19].

**Electronics**

Researchers, makers, and hobbyists who prototype with electronics rely largely on plastic parts to do so. The breadboards, enclosures, battery holders, buttons, and wires tend to all be made of materials that end up in a landfill [46]. Utilising mycelium provides a biodegradable alternative. These mycelium-based alternatives can be made of MBC blocks grown in moulds, which are then laser cut, laser engraved, or milled in order to create the grooves and housings needed to attach additional electronic elements and components. The fact that mycelium is not a conductor of electricity, and has a high fire-resistance rating, makes it beneficial for these materials to be used in applications where such characteristics are sought after. 

**Building components**

Using MBC as insulation capitalises on the inherent thermal and fire-resistance properties of mycelium-based material. The company Biohm’s mycelium insulation panels are able to achieve a thermal conductivity of 0.024 W/m⋅K This surpasses the values that can be achieved by market leading but unsustainable materials such as glass fibre (0.032–0.044 W/m⋅K), mineral wool (0.032–0.044 W/m⋅K), expanded polystyrene (0.036 W/m⋅K) and extruded polystyrene (0.029–0.036 W/m⋅K) [13]. Furthermore, the cost of manufacturing is low enough to compete with standard materials, which is a unique advantage in this implementation of mycelium.

The high insulative properties are also utilised in Elise Elsacker’s concrete slab system [17], introduced in Section 2.2.1 on subtractive manufacturing, which implements wire-cutting and mycelium blocks to cut a formwork for concrete that is left in place to become insulation. After the blocks are cut, beeswax is added to the surface of the mycelium to help prevent the concrete from fully penetrating the porous MBC shape. Any formwork not left in place is compostable, thus capitalising on the circularity of mycelium-based materials.

Another inherent material property of MBC is their favourable acoustical properties. The acoustical performance varies from product to product, but several companies and artists experiment with acoustical panelling systems made from mycelium-based materials. According to Pelletier et al. in “An evaluation study of pressure-compressed acoustic absorbers grown on agricultural by-Products”, an acoustical ceiling tile with a density of 0.71 g/cm^3^ and an MBC tile with a density of 0.42 g/cm^3^ have an acoustical attenuation of 7.6 and 7.1, respectively [67]. Therefore, the MBC panels are a comparable alternative to commercial acoustical tiles. 

Compressed mycelium panels are products that have yet to reach the consumer market, but they are a thoroughly researched variation of MBC. These panels are MBC that have been cold- or heat-pressed [4]. Once pressed, these materials become much denser as the air that once made the material light and airy is now lost in favour of a condensed material with improved structural properties. Cold-pressed MBC have a higher tensile strength and elastic modulus in comparison to non-pressed MBC, and result in a 2-fold density increase. Heat-pressed MBC further improve the tensile strength and elastic modulus, as well as having a more than 3-fold increase in density [30]. Compressed MBC, which have been heat-pressed, have panel-like characteristics and a compressive strength comparable to timber products such as oriented strand board (OSB) or medium density fibreboard (MDF). This is due to the softening of the lignin, which reacts to form new cross-links that increase the material strength [68]. In addition to the improved structural characteristics, heat-pressing also results in a decreased variation of density throughout panels and a consistent thickness. 

MBC-based floor tiles made by the Italian company Mogu consist of a topcoat of a water-based paint, a cover layer of 67% bio-based polyurethane with oyster shells, and a bio-composite core layer of 100% bio-based high density fibreboard [69]. The product is made of mycelium in addition to other materials, does not contain petroleum-based plastics, and incorporates recycled products. It is possible to biodegrade the tiles at the end of their lifecycle, so long as correct industrial conditions are provided. This serves as an example of a product in which mycelium is developed in a way that it can be used in combination with different materials.

#### 2.3.2. Architectural Projects

Architectural projects are constructions composed of more than one module of a mycelium-based material, or projects that are at a scale that is large enough to be considered an occupiable space. The following projects are categorised into in situ and prefabricated projects, as this differentiates between the fabrication techniques and geometries associated with either method of constructing with mycelium-based materials.

##### In Situ

**Shell Mycelium Pavilion (2016)**

The Shell Mycelium Pavilion by BEETLES 3.3 and Yassin Areddia Designs [70] used a triangular timber framework as the main structural geometry. The cavities of this geometry were filled with mycelium and then covered with coir pith consisting of coconut husk fibres. After some time, the top layer dried up and died, creating a protective layer over the growing mycelium. This project combined prefabricated modules and on-site assembly and growth of mycelium conducted in a non-sterile environment.

**Monolithic Mycelium Experiments (2017–2018)**

The monolithic mycelium experiments led by Jonathan Dessi-Olive [71] were a series of studies done to adapt mycelium to an in situ style implementation. Version 2 was an arch that used a waffle structure formwork made of laser cut cardboard onto which an inoculated substrate mixture was placed, which took over and inhabited the cardboard formwork to create an arch. The design of the arch implemented RhinoVAULT [72] to find a compression-only structure. The arch was allowed to grow for just under 5 days, and then the cover was removed to allow it to air dry for several weeks [71]. In the end, the small arch was able to carry the weight of a person and demonstrated both the importance of geometry and the possibility to grow in situ in a non-sterile environment.

**Voxel Bio-Welding and Jammed Bio-Welding (2019)**

This project was exhibited by David Benjamin of The Living on two occasions: in 2019 at the exhibition “Broken Nature” hosted by the Triennale of Milan, and again in 2019 at Centre Pompidou’s exhibition La Fabrique du Vivant [73]. On both occasions the projects showcased the creation of large-scale demonstrators using bio-welding as the dominant fabrication technique. The structure was composed of pre-compressed, loose granular blocks held in place with a temporary formwork system. The structure was inoculated and incubated in situ for a specific period. Finally, the formwork was removed and the substrate remained in place due to the binding action of the mycelium. 

**Tallinn Architecture Biennale Installation (2022)**

This project was a winning entry to the Tallin Architecture Biennale by Simulaa [74], which proposed a structure that grows as it is on display. Waste material from the local timber industry was harvested and combined with a biodegradable polymer, then 3D printed. The printed timber structure was then inoculated with the fungal strain that then consumes the structure and slowly replaces it over time until only the mycelium remains. The project prints the timber structure according to a generative algorithm using an industrial robot, illustrating the potential in combining new technologies with natural organisms [74].

##### Prefabricated

**Mycotecture (2009)**

This project is considered by many to be the origin of combining mycelium-based materials with prefabrication methods. The inventor-artist Philip Ross used a traditional arch shape and formed a relatively small construction that explored the relationship between people and grown architecture to expose people to the wonders of fungi [75]. The project used a small arch to create a relatively small, occupiable space. The bricks themselves grew edible mushrooms, which were used to brew tea for visitors [75].

**Hy-Fi (2014)**

The Hy-Fi pavilion by the Living was a 12 m tall pavilion that used 10,000 mycelium bricks of the MBC. These bricks were stacked and supplemented with an additional timber structure to stand. A limited number of moulds were used to produce the bricks, as the form was generated computationally to limit the number of unique moulds. The pavilion was left standing for three months. At the end of its life, all 10,000 bricks were composted and distributed to local gardens [76].

**MycoTree (2017)**

The MycoTree project is a collaboration between Karlsruhe Institute of Technology (KIT), Swiss Federal Institute of Technology (ETH) Zurich, Future Cities Laboratory in Singapore and Mycotech from Indonesia. This project utilises MBC grown in a mould in combination with connection made of laminated bamboo to create a load-bearing structure [4]. The moulds were digitally fabricated using readily available sheet material, and the overall design stems from the implementation of 3D graphic statics, a form-finding method for compression-only spatial structures [77]. The strength and stability of the structure are derived from its geometry, rather than the material. This project serves as a unique example of a structural implementation of MBC and demonstrates the advantages of integrating appropriate digital fabrication techniques with structurally informed computational design methods.

**The Growing Pavilion (2019)**

The Growing Pavilion was designed by set designer and artist Pascal Leboucq in collaboration with Erik Klarenbeek’s studio Krown Design at Amsterdam studio Biobased Creations [78]. The pavilion showcases several bio-based materials, such as wood, hemp, mycelium, cattail, and cotton, with the facade panels being made of mycelium. The fungal strain in these panels is *Ganoderma*, and the substrate is composed of aspen wood and hemp. This substrate comes from the residual flows of local farmers in the Netherlands [79]. This is a non-structural implementation of MBC and is a unique project in that the MBC are used as facade cladding with wood substructure.

**The Circular Garden (2019)**

The CRA (Carlo Ratti Associati), in partnership with the global energy company Eni, developed this temporary installation for the Milan Design Week in 2019 [80]. The installation was grown over a two-month period to create a series of 60 4 m tall arches. The geometry of these arches was based on an inverted catenary curve to be a compression-only structure. If added up, the arches would be nearly 1 km tall. The arches compose a series of four architectural “open rooms” throughout the garden [80]. This project combined monolithic mycelium structures with prefabrication techniques, highlighting a new approach to MBC.

### 2.4. Summary

Most of the project-scale implementations of mycelium show a strong trend toward using MBC grown in moulds. In most cases, the structure is discretised into smaller components to be prefabricated in a controlled environment and assembled on-site. In these projects, MBC are then used as non- or semi-structural components, requiring an exoskeleton or an auxiliary structure to provide the main stability. This can be seen in the wood frame structure of the Hy-Fi pavilion and the Growing pavilion. MycoTree is a unique project in which mycelium is used in a load-bearing capacity, achieved through the unique geometry of the structure. However, the height of the structure was a major limiting factor due to the low mechanical properties of MBC. 

In projects where the mycelium is grown on site, the size of the projects is considerably smaller due to logistical difficulties and the long duration associated with the growing of mycelium in exposed environments. Additionally, the inadequate material strength fails to accommodate the increasing self-weight as the size of the structure increases. As a result, mycelium-based material applications that have had the most success in reaching the construction industry are thermal or acoustical insulation panels. These applications demonstrate that the jump from product to project has not yet seen success in becoming a marketable construction method. 

While the compostable nature of mycelium-based materials is an advantage in terms of circularity, it can also present several challenges. While these materials themselves are entirely compostable, some MBC packaging materials take only approximately 40 days to biodegrade [81]. This means that these materials can degrade quickly over time, which reduces their shelf life.

## 3. Challenges in Scaling up Mycelium

The following challenges are based on a review of the advantages and disadvantages of the previously discussed products and projects. Opportunities for addressing these challenges are speculated and proposed, and how they could broaden the potential of mycelium-based materials in large-scale architectural applications is discussed.

### 3.1. Structurally-Informed Design

The primary reason that mycelium-based materials have not been used as load-bearing elements is their inadequate structural properties. For example, the Living implemented a method of stacked bricks to construct the Hy-Fi pavilion. This construction technique is historically combined with masonry, as it is a relatively simple yet effective way to construct. However, according to Granta CES EduPack, a common clay brick has a compressive strength of 69–140 MPa [82], while MBC grown in a mould have a compressive strength of 0.35–0.75 MPa [83]. This shows the difference in strength between these two materials and highlights how these construction techniques for conventional materials are not appropriate or directly applicable to the construction of large, self-supporting structures made of mycelium-based materials. 

The development and engineering of conventional materials such as MET, steel, and concrete are largely based on achieving material strength by increasing their allowable stresses. Although mycelium-based materials have numerous ecological benefits including repurposing of waste streams, biodegradability, and recyclability at end-of-life, their low structural strength significantly limits their applicability in large-scale products and projects. In order to scale up structural applications of materials with low tensile and bending strength, the geometry of the structure plays an important role in activating the material in compression only. With appropriate computational design tools, stability can be achieved through geometry rather than material strength. This allows relatively weaker materials such as mycelium to be used structurally and safely in large scale architectural applications. While this does not indicate that a multi-story masonry hi-rise can be constructed with mycelium with compression-only geometries, it does indicate that these materials can achieve a higher potential if given their own architectural vernacular according to their inherent properties to achieve small-to-medium scale constructions.

### 3.2. Region-Specific Material Profiles

Traditional materials such as steel, wood, and concrete have standardised mechanical properties and established industry regulations that are useful resources for architects and engineers. The disparity in material characteristics between different mycelium-based materials is in large part due to their inherent specificity to the given context and how the research on these materials is conducted in different regions across the globe. While most fungal strains–typically *Ganoderma lucidum*, *Pleurotus ostreatus*, or *Trametes versicolor*–are readily available across various geographic regions, the agricultural waste streams that are typically used as substrates are highly dependent on cultural, urban, and climatic conditions of the regions. For example, while rice husk is a common substrate used in mycelium-based materials for countries located in Asia [84], this waste material is not as common in Europe. This leads to material compositions with the same fungal strains but different substrates, and thus to divergent material properties. The relationship between fungal species and type of substrate is one of the essential factors affecting the research around mycelium-based materials. The customisability of recipes and production processes can result in different material characteristics (i.e., foams, sandwich composites, etc.) and products that are specifically tailored to the unique geographical and climatic constraints, as well as region-specific building regulations. This condition opens new research questions concerning:

- Identification and selection of nutritive and non-nutritive substrates available in a specific region in relation to their sector of origin (construction, agriculture or urban by-product);
- Determination and characterisation of nutritional profiles of substrates;
- Investigation, comparison, and classification of new fungal species potentially dominant in a region; and
- Investigation of genetic engineering to maximise the performance of region-specific recipes for mycelium-based materials.

Understanding the inner dynamics of interfacial bonding between substrates and fungal species may help to create new mycelium-based materials with more carefully customised properties such as the material strength, acoustical performance, thermal values, etc. For example, the more difficult it is for the mycelium to digest the substrate, the higher is the stiffness of the composite [85]. As research on mycelium-based materials expands into more regions with different substrate options, the generalisation of the relationship between substrates and growing conditions to material properties will aid in both the development of regional profiles and in a generalised understanding of the connection between fungal strains, substrates, and material properties. 

### 3.3. Identification of Viable Waste Streams

In addition to the selection of substrate types, identifying sizable waste streams that are suitable as substrates for MBC production remains challenging. In order to enable and support circularity within the construction industry, the repurposing of construction waste would be optimal. However, the presence of additives such as synthetic adhesives and other chemical treatments involved in the production of natural materials such as MET makes it challenging to find suitable alternatives from the construction industry to waste from agricultural and culinary industries. Specifically in the commercialisation of mycelium-based materials, chemical treatments applied post-fabrication are currently a factor affecting the re-use and compostability of the material, as seen in the Mylo product. 

Another key factor to consider is the creation of waste during the manufacturing process. While using prefabricated moulds as the dominant method of fabrication for MBC simplifies the manufacturing process on a larger scale, it can also create new waste streams if not taken into consideration when designing the overall manufacturing workflow. 

### 3.4. New Mycelium Typologies

Typically, different typologies of mycelium-based materials are used in isolation from one another and are often combined with materials such as wood. However, there is a unique opportunity in exploring applications that combine the strengths of different material typologies. For example, PMM is a potential resource for laminating lignocellulosic materials together [86], while MBC, when left alive, can grow a thickened skin and join together separate blocks as shown in this mock-up installation [87]. Combining mycelium-based material typologies could potentially lead to the creation of new bio-composites with improved thermal, acoustic, and mechanical properties that cannot be achieved by a single typology alone.

### 3.5. Industrialisation of Biological Processes

The field of mycelium-based materials is currently confronted with two different tendencies: (1) the one dictated by the technological paradigm of automation, and (2) the one created by the use of biotechnologies in the built environment. Specifically, the renewed interest and development in biotechnologies applied to the built environment foresees a new material culture that can potentially overcome the separation between industrial processes and natural environmental cycles. The technologies and research developed within the context of mycelium-based material research, especially when confronted with applications on a large scale, come with a series of challenges that converge with the emergent field of construction biotechnologies. This new emergent field introduced by Ivanov and Stabnikov [88], investigates new microbial-mediated construction processes and microbial production of construction materials. Despite the advancement, the chemical, manufacturing, and construction industries remain somewhat reluctant to adopt bio-based or bio-inspired practices that could replace current oil-based processes. Both civil and environmental engineering are conspicuously developing a variety of solutions taking into consideration fundamental knowledge of microbiology. However, this type of knowledge is often a general case of “applied biological science” rather than firmly weaved and developed within design and engineering problems. In this regard, there are three main considerations to consider when scaling up bio-based material systems. 

**Scalability**

Manufacturing process for new materials with high impact and a growing market value comes with an inherent potential for large-scale industrialisation. The case of mycelium-based materials shows a biological process (similar to other industrial fermentation processes, such as cheese, beer, etc.) characterised by various complex variables that include long cycles of production, risk of contamination, and a complex multi-step manufacturing process. The lack of consistency and reproducibility of a specific material profile, therefore, prevents the scaling up of mycelium in both size and quantity to reach an industry-standard level of product certification. Companies such as Ecovative and Biohm have started to address some of these considerations using genetic engineering, optimisation of fungal strains, and introduction of additional micro-organisms in the fabrication loop.

**Reproducibility**

One of the key factors facilitating the development of new materials in engineering disciplines is the adoption of a comprehensive and reliable set of standards. Such levels of standardisation are difficult to achieve in the context of biological systems, which often have a high degree of uncertainty. This condition does not refer exclusively to the complexity of the biological systems themselves, but to the availability of infrastructures that have the capacity to support the development of standardised practices. Specifically, one of the main limitations to overcome is the conversion of the laboratory scale protocols into much larger and industrial-size equivalents. 

**Automation**

The use of machines and new manufacturing technologies in highly specialised and controlled environments is an important challenge shared by industries ranging from biomedicine, chemistry, and biotechnology to architecture. Weariful and repetitive tasks that are prone to error when done by humans are already being accomplished in many laboratories and industries with robots. This includes tasks such as liquid handling, as well as additive and subtractive manufacturing. The use of machines in environments such as the ones needed to produce mycelium-based materials creates a condition that intrinsically minimises human intervention, ensuring a safe environment during the fabrication process.

### 3.6. Agile In Situ Setup and Applications

In large part, mass-produced mycelium-based materials that have seen commercial success consist of MBC grown in moulds. This fabrication method relies on a closed, sterile environment. Growing mycelium in situ without a controlled environment has only seen success on a small-scale, while 3D printing has similarly succeeded on a small scale and within a controlled environment. Scaling up in situ applications requires a better understanding of preventing contamination, as well as the development of techniques that do not rely on such a restrictive, closed-off space. Whereas prefabricated MBC can rely on moulds to ensure a closed environmental system, when scaling up there is a need for this controlled environment to be larger than just the mould of one module.

Implementation of new fabrication techniques with mycelium-based materials in a controlled, sterile environment at an architectural scale presents a number of challenges. For example, the use of a robotic arm for mycelium prototyping requires a cleanroom with accurate humidity and temperature control. This constraint is an opportunity to create a walk-in prototyping space able to facilitate the production of large-format prefabricated and highly customised parts. These environments can potentially be located adjacent to the construction site, ultimately reducing costs of transport. 

Within this framework, it may be even possible to consider these controlled environments as new construction sites, wherein prefabricated parts and substrates are simply installed and subsequently bound together using the binding capacity of the mycelial network. By approaching the controlled environment as the construction site, it is possible to develop an in situ bio-assembly process in which building parts are naturally bound and mechanically enhanced.

### 3.7. Cross-Disciplinary Research

The relevance of mycelium-based materials, as demonstrated in this paper, is not limited to their original fields of microbiology and mycology. Thanks to the work of researchers across many disciplines, their relevance has expanded into architecture. These fields are practically unrelated by tradition and in practice. However, the research and development to explore bio-based materials in general have drawn them closer together. While this increases the scope of research and development being done with these materials, it also means that this information does not easily cross over into the other fields. Furthermore, whenever that information does make the jump between disciplines, it can be difficult for an architect, for example, to discern the findings of a microbiologist. Therefore, the cohesive understanding of latest findings and material developments presents its own challenge as many factors prevent the knowledge from being distributed among experts from different industries.

As research on mycelium-based materials continues to exponentially increase year after year, the accessibility of the research also increases. This indicates that the visibility of research increases, and thus the dissemination of information across fields of research will naturally increase. Active efforts to regularly collaborate across different industries can best promote the sharing of findings and development of new technologies. The conscientious integration of the advances from different fields can also further unify the industries and spur new research. In this way, several other previously mentioned challenges in scaling up applications of mycelium-based materials can be addressed, such as the generalisation of how substrates, fungal strains, and environmental conditions affect the growth of mycelium. A further integrated approach can also begin to establish new, open-source practices for goals such as mass-production to expedite the scaling up of mycelium-based materials.

## 4. Conclusions

In this paper, we reviewed the latest advancements in mycelium-related research and applications from academia and industry to understand the diversity of perspectives and advancements. The advantages of using mycelium-based materials in the construction industry can incentivise the birth of new protocols, consider waste as a resource, and develop new methodologies to evaluate and assess the impact of the built environment. Thus far, mycelium-based materials have been most successfully implemented at the scale of small commercial products and commodities, with a number of challenges barring the expansion of applications to architecture. Coupling a material such as MBC with appropriate computational design and digital fabrication methods, while also developing mass-production techniques and local material profile strategies, has the potential to successfully scale up architectural applications of mycelium-based materials. The challenges and research gaps we identified are cross-disciplinary in nature and will require a close collaboration on material research, integrated design methods, appropriate digital fabrication techniques, and on-site implementation strategies from microbiologists, material scientists, manufacturers, and designers alike.

## Data Availability

Not applicable.
